# Molecular Alterations Associated with Osteosarcoma Development

**DOI:** 10.1155/2012/523432

**Published:** 2012-02-15

**Authors:** Kosei Ando, Kanji Mori, Franck Verrecchia, Baud'huin Marc, Françoise Rédini, Dominique Heymann

**Affiliations:** ^1^INSERM, UMR-S 957, 1 Rue Gaston Veil, 44035 Nantes, France; ^2^Physiopathologie de la Résorption Osseuse et Thérapie des Tumeurs Osseuses Primitives, Université de Nantes, Nantes Atlantique Universités, EA3822, 1 Rue Gaston Veil, 44035 Nantes, France; ^3^Equipe Labellisée Ligue 2012, Nantes, France; ^4^Department of Orthopaedic Surgery, Shiga University of Medical Science, Tsukinowa-cho, Seta, Otsu, Shiga 520-2192, Japan; ^5^Nantes University Hospital, Nantes, France

## Abstract

Osteosarcoma is the most frequent malignant primary bone tumor characterized by a high potency to form lung metastases which is the main cause of death. Unfortunately, the conventional chemotherapy is not fully effective on osteosarcoma metastases. The progression of a primary tumor to metastasis requires multiple processes, which are neovascularization, proliferation, invasion, survival in the bloodstream, apoptosis resistance, arrest at a distant organ, and outgrowth in secondary sites. Consequently, recent studies have revealed new insights into the molecular mechanisms of metastasis development. The understanding of the mechanism of molecular alterations can provide the identification of novel therapeutic targets and/or prognostic markers for osteosarcoma treatment to improve the clinical outcome.

## 1. Introduction

Osteosarcoma (OS) most often occurs, during childhood and adolescence, in the metaphysis of long bones, including large growth plates with high proliferation activity and bone turnover [1]. Historically, patients with primary OS have been treated with resection surgery alone, resulting in poor prognosis. Clinical outcome of localized OS has improved with neoadjuvant chemotherapies, based on methotrexate, cisplatin, doxorubicin, and ifosfamide treatments. The 5-year survival has indeed increased to around 60%. However, the 5-year survival of patients with OS metastasis still remains about 30% [2–7]. OS metastases appear most frequently in the lung [8] and are the main cause of death for patients with OS, because micrometastases are undetectable at initial diagnosis [9, 10]. Taken together, OS patients with metastases present further worse clinical results than those without metastases. Thus, more effective treatments and/or a more personalized therapy (i.e., treatments according to specific genes or protein profile expressions) are needed for patients with OS associated with pulmonary metastases.

**Figure 1:**
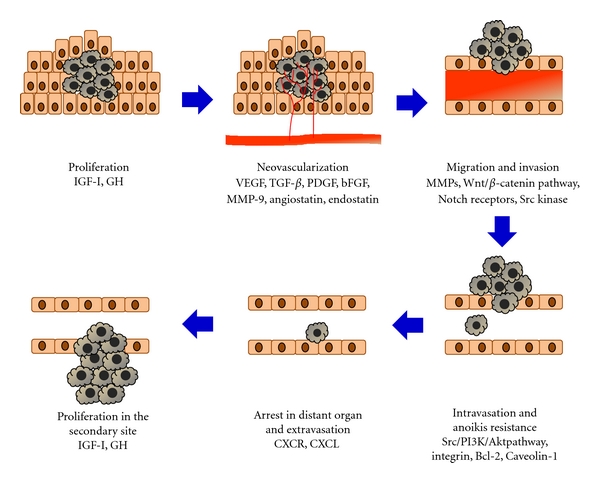
The main steps of the tumor metastatic process. Tumor cells proliferate at the primary site and neovascularization is induced by tumor environment such as hypoxia. In turn, they migrate and invade into the bloodstream. These tumor cells in the circulation need to survive against anoikis to arrest in a distant organ. Metastatic colonization at the secondary site involves the interactions between tumor cells and the microenvironment.

The establishment of cancer metastasis involves several complex steps: intravasation, survival in the circulation, arrest at a distant organ, extravasation, and growth in secondary sites (Figure 1). Molecular alterations of these steps have been practically analyzed. The understanding of metastasis mechanism might allow us to find new molecular targets for improvement of the patients' survival. This paper describes the molecular factors associated with OS development and summarizes the main molecular alterations involved in this bone disease, especially in metastatic OS, which strongly contribute to the development of novel therapeutic approaches.

## 2. Neovascularization is a Key Parameter in Osteosarcoma Growth

Nutriments and oxygen required for the metabolism of normal and tumor cells are delivered by blood vessels. Neoformation of blood vessels allows growth, invasion, and metastatic spread of cancer cells in malignant pathologies. [11, 12]. The process of neovascularization is generally regulated by a balance between angiogenic inducers and inhibitors. The shift in favor of angiogenic inducers, known as the “angiogenic switch,” promotes the formation of a new blood supply enhancing tumor growth and metastasis. Neovascularization is induced by the tumor environment such as hypoxia, acidosis, or inflammation in an oncologic context. In these conditions, both tumor cells and host endothelial cells can increase the expression of proangiogenic: vascular endothelial growth factor (VEGF), platelet-derived growth factor (PDGF), basic fibroblast growth factor (bFGF), and transforming growth factor (TGF-*β*) [13–17]. Tumor cells also secrete proteolytic enzymes such as matrix metalloproteinases (MMPs), which degrade basement membrane and extracellular matrix (ECM) promoting cell dissemination [18, 19]. MMP-9 is indeed highly related to the angiogenic switch because it can activate proangiogenic factors [20, 21]. Several studies have demonstrated that VEGF or TGF-*β* expression is associated with an increase of tumor vascularity, invasion, and poor prognosis in OS [22–24]. It has been shown that high serum-VEGF levels in OS correlate with tumor progression, metastasis, and poor prognosis [25, 26]. However, the relationship between an increase of tumor vascularity and a poor prognosis is controversial in OS [27–29].

The well-known angiogenic inhibitors are angiostatin and endostatin. Angiostatin is a cleavage product of plasminogen [30], whereas endostatin is the carboxyl-terminal fragment of collagen XVIII [31]. They inhibit endothelial cell proliferation and migration [32]. The resulting antiangiogenic activity has been demonstrated in various tumor models *in vivo* [33–38]. Based on these (pre)clinical results, clinical trials are currently running to evaluate the effect of human recombinant endostatin. Although showed a well tolerability and safety in patients with malignant solid tumors, it induces a minor antitumor effect not related to the vascular changes [39–41]. Inhibition of neovascularization should suppress tumor growth despite tumor cell heterogeneity because blood supply is necessary for all tumors to survive. Furthermore, the available data from animal models and phase I and II clinical trials of angiostatin and endostatin have shown that these agents are well-tolerated at therapeutic doses: 15–600 mg/m^2^/day added to those patients, although the use of antiangiogenic therapy has raised the debate about interference with normal physiological processes such as wound healing and tissue repair [31, 39–43].

## 3. Migration and Invasion: Two Potential Therapeutic Targets

Tumor migration and invasion through the ECM are critical in metastatic dissemination [15, 16]. Degradation of the ECM, which leads to migration, invasion, and metastasis, releases MMPs (MMP-2 and MMP-9, in particular) and m-calpain in OS [44–46]. In addition, the Wnt/*β*-catenin, Src-kinase and Notch signaling pathways are also involved in migration and invasion [47–55].

MMPs are a family of zinc endopeptidases consisting of at least 20 different members and regulate different cellular metabolic processes [56, 57]. They induce a variety of biological effects including growth, morphogenesis, apoptosis, tissue destruction, and cancer formation [58, 59]. Recently, bisphosphonates have been shown to downregulate MMPs expression and reduce the invasive potency of OS cells [60–64]. Disulfiram is also able to control the invasion and metastasis in human OS cells through the MMP-2 and MMP-9 inhibition [65]. Both of m-calpain expression and MMP-2 secretion are inhibited by a siRNA targeting m-calpain in SAOS-2 cells [46]. m-calpain is also essential in the invasion and human OS metastasis [46]. These agents related to proteases represent new therapeutic targets and approaches to decrease the OS migration and invasion.

Wnt signaling pathway coordinates osteoblast proliferation and differentiation [66]. Disruptions in various components of the Wnt pathway result in disordered bone development and homeostasis [67]. The *β*-catenin-dependent Wnt signaling pathway is regulated by secreted Wnt antagonists divided into two groups. Wnt inhibitory factor 1 (WIF-1) and the secreted frizzled-related protein family directly bind to Wnt ligands while the Dickkopf families and sclerostin are blocking Wnt receptors trough the endocytosis of low-density lipoprotein receptor-related protein 5/6 coreceptors [68–71]. This Wnt binding leads to the activation of disheveled, which in turn, releases *β*-catenin from the axin-adenomatous polyposis coli-glycogen synthase kinase-3*β* complex, causing stabilization and accumulation of *β*-catenin in the cytoplasm. After its translocation to the nucleus, *β*-catenin binds to the T-cell factor/lymphocyte enhancer factor family of transcription factors and promotes downstream target oncogenes such as c-myc, cyclin D, survivin, and MMPs. These mechanisms are involved in proliferation, invasion, and metastasis in various human cancers [72–75]. OS frequently expresses high levels of cytoplasmic and/or nuclear *β*-catenin [76], which is also associated with metastasis [77, 78]. These findings suggest that aberrant Wnt activation is crucial in multiple cancers, including OS [79–81]. A preclinical study has demonstrated that the inhibition of Wnt/*β*-catenin pathway induced lower levels of nuclear *β*-catenin, resulting in downregulation of the *β*-catenin-targeted genes such as MMP-9, cyclin-D, c-myc, and survivin [82]. Several reports have demonstrated that WIF-1 silencing due to hypermethylation results in Wnt signaling activation in a variety of cancer. WIF-1 can inhibit the cell growth of those cancer cells [79, 80, 83–86]. The downregulation of WIF-1 expression plays a role in OS progression. Reexpression of WIF-1 also suppressed Wnt signaling pathway, resulting in the tumor growth and lung metastasis *in vivo* in OS mouse models [50]. These results indicate that WIF-1 can be a therapeutic agent against OS metastasis. However, the function of Wnt antagonists including WIF-1 is still unclear and further investigations are needed.

Notch signaling regulates development of many tissues and cell types through diverse effects on cell fate decision, stem cell renewal, differentiation, survival, and proliferation [87]. Notch signaling is one of several evolutionarily conserved signaling pathways in the development of multicellular organisms. Its temporal-spatial expression effects can specify diverse cellular events, including proliferation, differentiation, apoptosis, and stem cell maintenance. In mammals, there are four Notch receptors: Notch1-4, and eleven ligands [88]. The first targets of Notch are two basic helix-loop-helix transcriptional repressor families: the Hairy/enhancer-of-split (Hes) and the Hes with YRWP motif families [89]. Notch has been considered as a promoter of invasion in OS. The Notch receptor 1, 2, and Hes1 genes induced by Notch increase in highly metastatic OS. The Hes1 gene was inversely associated with the survival rate in human OS [52–55]. The OS cell invasion was reduced by an inhibition of the Notch signaling pathway whereas the cell proliferation was not blocked in a preclinical setting. The Notch-inhibited cells were less able to induce lung metastases in an orthotopic mouse than the negative controls. However, the mechanism in the inhibition of the Notch pathway and the downregulation of invasion resulted from Hes1 remains not clear [53, 55].

Src is a nonreceptor tyrosine kinase and encoded by the c-Src as a protooncogene. Src kinase activity is regulated by several receptor tyrosine kinases (RTKs) such as epidermal growth factor (EGF) RTK, PDGF-RTK, and integrin receptors [90–92]. Src family kinases are critical in the metastatic dissemination, such as cell proliferation, adhesion, invasion, survival, and angiogenesis. Either overexpression or activation of c-Src has been shown to occur in cancer development [49]. Src, involved in tumor metastasis widely, could be a novel therapeutic target in OS metastasis. Dasatinib, known as a Src kinase inhibitor, suppresses Bcr-Abl tyrosine kinase. The effect and safety of dasatinib have been established as therapeutic agent for imatinib-resistant chronic myelogenous leukemia in early-phase clinical trials. Also, several studies have shown that the dasatinib acts against Bcr-Abl-positive leukemic cell lines as well as other malignancies. The c-Src-mediated signaling pathways, related to tumor proliferation, adhesion, or migration, have been shown in various malignancies such as prostate cancer, lung cancer, and sarcoma [93–95]. In preclinical studies, dasatinib suppressed tumor migration and invasion with inhibition of the Src kinase activity and its downstream signaling in OS cell lines *in vitro* [48, 96]. On the other hand, dasatinib had no effect on pulmonary metastases *in vivo *[48]. At present, the other specific Src kinase inhibitor, called saracatinib, is under investigation in phase II clinical trial of OS lung metastases (clinicaltrials.gov/ct2/show/NCT00752206).

## 4. Apoptosis Resistance and OS Progression

Apoptosis is involved in cell survival in cancer metastasis through the all stages *via* two pathways. The first one is regulated by a death-receptor-bound to Fas or tumor-necrosis factor (TNF) family member, death-inducing signaling complex, and caspase-8. The second one is associated with p53, Bcl2 family member, cytochrome-c, and caspase-9. When caspase-8 or -9 is activated, caspases of the downstream can be cleaved inducing cell death. Fas and its ligand (FasL) belong to the TNF death receptor superfamily and regulate tumorigenesis in a variety of primary malignancies and metastases [97–99]. Fas/FasL complex, constitutively expressed in lung tissue, enhances the Fas-apoptosis pathway and leads to cell death [100, 101]. Fas receptor has been well known as a death receptor mediated apoptosis in a variety of tumor cells. Recent studies have revealed that Fas is also proapoptotic related to tumor proliferation, differentiation, and migration [102–104]. Thus, apoptosis resistance is crucial for establishment of tumor metastasis; it is implicated in treatment resistance with cancer metastasis [105]. Fas expression is often decreased in OS lung metastasis, whereas it is highly expressed in the primary tumors [100, 101, 106]. Furthermore, Fas-negative expressions correlate with tumor development and poor prognosis [100, 101, 107–109]. Inhibition of Fas signaling and/or the loss of FasL can develop the proliferation of Fas-positive OS cells in the lungs and can promote the growth of lung metastases in OS models *in vivo* [107].

Interleukin- (IL-) 12 increased the expressions of Fas receptor in OS lung metastasis through stimulation of the Fas promoter activity. In turn, the metastatic cells acquired the susceptibility to FasL in relation to Fas-induced apoptosis in the lung microenvironment [110]. *In vivo*, combination therapy of IL-12 with ifosfamide induces FasL expression, increasing the therapeutic efficacy via the Fas/FasL pathway [111]. Muramyl tripeptide phosphatidyl ethanolamine (MTP-PE) induces IL-12 production in OS patients through activation of macrophages [1, 112]. MTP-PE also up-regulates Fas expression when exogenous IL-12 is administered to the patients [106]. The combination of MTP-PE with ifosfamide induces IL-12 and FasL, respectively, consequently the clinical outcome of the treated patients can be improved through the activation of tumor apoptosis [106]. These results suggest that Fas death receptor pathway may enhance the efficacy of chemotherapy in OS.

IL-18, which is an interferon-*γ*-inducing factor [113], affects an antitumor effect via the activation of natural killer (NK) cells or cytotoxic T cells [113, 114], inhibition of angiogenesis [115] and induction of FasL on Fas-positive tumor cells [116]. IL-18 has been shown to inhibit metastasis in OS cells through the activation of T-cells and NK-cells and the induction of the FasL expression [117]. In addition, the combination of ifosfamide with IL-18 suppresses the development of OS lung metastasis [118]. Taken together, Fas death receptor pathway is essential in the establishment of OS lung metastasis, and it may be a novel therapeutic target. However, the molecular mechanism of the loss of Fas-mediated apoptosis in OS metastases is unknown.

## 5. Survival in the Blood Circulation: Anoikis Resistance

Cancer metastases require the anoikis-resisted cells to survive in the circulation. Anoikis, Greek for “homelessness,” regulates cell homeostasis in tissues. Normal epithelial cells become apoptotic when exposed to anchorage-independent environments [119, 120]. In turn, once tumor cells have entered into the bloodstream to disseminate distantly, the cell-cell adhesions or ECM attachments are lost, which results in the specific apoptosis called anoikis [121]. Therefore, metastatic cells need to acquire the resistance to anoikis to survive during dissemination and colonization of secondary distant sites in the circulation.

Acquisition of anoikis resistance has been described in nonepithelial malignancies such as OS [122]. Many studies demonstrated the survival mechanism of cancer cells in the evasion of anoikis with various means such as Src/PI3K/Akt pathway, focal adhesion kinase, or Bcl-2 [123, 124]. Several studies have shown that *β*4 integrin expression is involved in cancer progression [125–127]. The *β*4 integrin expression is also implicated in the survival of OS cells in the circulation, because knockdown of *β*4 integrin suppressed the cell-proliferation under anchorage-independent sites in OS cells [128]. In addition, the knockdown of *β*4 integrin in a mouse model inhibited lung metastases, and *β*4 integrin-ezrin interaction appears to be essential for *β*4 integrin expression. However, the relation between ezrin and *β*4 integrin is still unknown [128]. Cell-cell adhesions can activate integrin signaling in anchorage-independent conditions and integrin expression patterns may contribute to the resistance to anoikis [129].

Switch from *α*_V_ *β*_5_ to *α*_V_ *β*_6_ integrin may suppress anoikis in squamous cell carcinoma cells through the activation of PI3K/Akt signaling pathway [130]. The PI3K/Akt pathway, which depends on Src kinase activation, is important for human OS cells to avoid anoikis [47]. Src has another role related to anoikis resistance with caveolin-1 in OS cells. Caveolin-1 is the major protein component of caveolae [131], which regulates several intracellular signaling pathways [132]. Caveolin-1 is highly expressed in osteoblasts [133] and its overexpression in OS cells inhibited anchorage-independent growth, invasion, and migration by blocking c-Src and c-Met tyrosine kinases* in vitro *[134]. In addition, Caveolin-1 overexpression suppressed the OS metastasis *in vivo *[134].

## 6. Arrest and Extravasation: Final Step of Cell Migration

The mechanism of migration arrest of metastatic cells is controversial. Metastatic tumor cells are generally thought to be trapped in the microcirculation because their size is larger than that of normal cells [135]. When the tumor cells in the bloodstream are trapped, microembolisms are structured, and the interaction with the local microenvironment begins consequently. Interestingly, cancer cells have the tendency to prefer a specific target organ in metastasis processes: Over 80% of all metastases in OS occur in the lungs [136]. This result suggests that circulating tumor cells can select their optimal sites to survive and grow via interactions with distinct molecules expressed on the endothelial cells in the distant organs [16]. In the circulation, cell colonization in the distant organs is mediated through the secretion of chemokines and proteinases, involved in extravasations [15, 16]. Recently, chemokines are regarded as important factors to control a site specificity of cancer metastasis including OS-lung relation [137–140]. C-X-C-motif chemokine receptor 4 (CXCR4) and its ligand C-X-C-motif chemokine ligand 12 (CXCL12) have been shown to regulate an organ-specific metastasis by the formation of chemotactic gradients in several cancer [141–143]. Binding of CXCR4 to CXCL12 allows adhesion and extravasation of OS cells in pulmonary metastasis [138, 139, 144, 145]. These results suggest that abundant expressions of CXCL12 in the lung may be involved in the high frequency of pulmonary metastases in OS. Highly CXCR4 expressions in OS-patient samples adversely correlated to event-free, overall, and metastasis-free survival [138]. These data suggest that CXCR4 could be useful as a prognostic factor in OS metastasis.

CXCR3, another chemokine receptor, has been identified in a variety of malignancies including OS [138, 146–148]. Its ligands, CXCL9, 10, and 11, are expressed in lungs. The inhibition of CXCR3 chemokine pathway down regulates the growth of OS lung metastasis. Recent study has demonstrated that CXCR3 inhibitor decreased the proliferation, survival and invasion of the tumor cells in an animal model of OS lung metastasis. In other words, the interaction between CXCR3 and its ligands can directly enhance the invasion, survival, and proliferation of tumor cells in the metastatic organ. This result suggests that targeting CXCR3 can specifically inhibit OS lung metastasis [144].

## 7. Adhesion Step in the Metastatic Process

Establishment at a distant organ requires that the metastatic cell connects to its new environment and re-establishes cell-cell adhesions. Ezrin is a membrane-cytoskeleton linker protein that acts as membrane organizers and linkers between plasma membrane and cytoskeleton controlling cell-microenvironment and cell-cell interactions [149]. In addition, ezrin associates with several signaling transductions, such as Rho and PI3K/Akt pathways [150, 151]. Recently, high level expression of ezrin protein is correlated to metastasis in several cancers [152–154] as well as OS [155, 156]. High expression of ezrin is associated with pulmonary metastasis in animal models [155, 157], and with poor outcome in pediatric OS patients [155]. Phosphorylated ezrin was shown to express at just early phase in lung metastasis [155] whereas it was dynamically expressed at both the early and late time point [156].

Sorafenib is a multipotent drug, and several molecular targets of sorafenib such as Raf kinases are implicated in OS development [158, 159]. Recent preclinical study has reported that sorafenib suppressed the development of lung metastases via downregulation of ezrin-activated mitogen-activated protein kinase (MAPK)/Akt signaling [160]. In addition, sorafenib could induce apoptosis through a decrease of expression of the antiapoptotic Bcl-2 family [160]. These data suggest sorafenib may be a novel potential therapeutic option in patients with OS metastasis.

## 8. Main Signaling Pathways Involved in Proliferation of Metastatic OS

OS pathogenesis is clearly related with bone growth during adolescence, suggesting a potential relationship with higher expression of hormone levels [161, 162]. Thus, several studies have suggested that molecular alterations in the growth hormone (GH)/insulin-like growth factor I (IGF-I) signaling pathways could lead to OS development *in vitro* and *in vivo *[163, 164]. OS cells show both IGF-I and IGF-I receptor expression and highly response to IGF-I *in vitro *[164]. Serum IGF-I levels in mice with hypophysectomy are significantly downregulated, which is decreasing tumor growth and development of metastasis [165].

A phase I trial in patients with metastatic and/or recurrent OS was performed with somatostatin analog (OncoLar) to reduce serum IGF-I [166]. In this trial, OncoLar treatment in 21 OS patients resulted in a 46% decrease in serum IGF-I levels without toxicity. In a preclinical study conducted on dogs with naturally occurring OS, OncoLar [167] reduced serum IGF-I levels were by approximately 43% without toxicity. However, no difference in primary tumor necrosis, apoptosis, or survival was observed in dogs treated with a combination of OncoLar and chemotherapy in comparison with just chemotherapy. These observations indicate that the extent or duration of serum IGF-I suppression induced by OncoLar was not enough to improve a clinical outcome. IGF-I receptor (IGF-IR) axis is also implicated in OS development; inhibition of IGF-IR could inhibit tumor growth, activate apoptosis and up-regulate the chemosensitivity and radiosensitivity in OS cells [168, 169].

Recently, human monoclonal antibodies targeting the IGF-IR were tested in both preclinical and clinical studies. Inhibition of IGF-IR with some monoclonal antibodies enhances the antitumor effects in several OS xenograft models [170, 171]. More recently, a clinical study has demonstrated that high IGF-IR expression is a poor prognostic factor for OS patients leading to OS development and metastasis [172]. Thus, IGF-IR targeting therapy can be a novel strategy for the treatment of OS associated with metastasis.

## 9. Dormancy

Unfortunately, tumor metastasis occasionally occurs for patients with malignancies a long time after the success of primary therapy [173, 174]. This latency period is generally the result of tumor dormancy, which is frequently asymptomatic and clinically undetectable for months or years until relapse. Once tumor cells are settled in a secondary site, they can grow, die by apoptosis, or remain dormant. Two ways of tumor dormancy have been described, (i) tumor mass dormancy (dormant micrometastases) and (ii) cellular dormancy [174–176]. In dormant micrometastases, tumor cells generally divide but the growth is limited. Cellular dormancy (dormant single tumor cell) can occur when tumor cells enter in a quiescence state and do not divide any more. Tumor cells in dormancy are usually resistant to conventional drug because current treatments target cells in division. However, the mechanisms allowing dormant tumor cells to survive to conventional chemotherapies and then resume the tumor outgrowth remain unknown.

**Figure 2:**
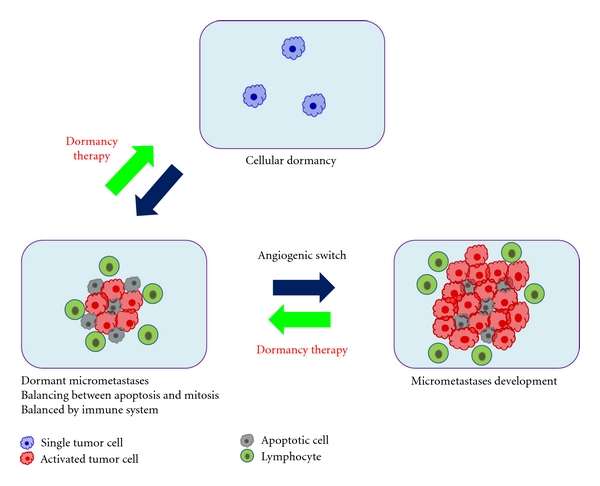
Tumor metastasis dormancy is associated with the risk of recurrence of OS and late development of lung metastases. Tumor dormancy is thought to consist of tumor mass dormancy (dormant micrometastases) and cellular dormancy. In tumor mass dormancy (dormant micrometastases), tumor cells generally divide but not in cellular dormancy. The tumor growth is strictly limited by the lack of blood supply or immune system. Dormant state of micrometastases is involved in angiogenic switch and/or the adaptive immune system. Dormancy therapy could contribute to improve the treatment of patients with cancer.

Dormant micrometastases are thought to be present under a balance between cell proliferation and apoptosis [176, 177]. Dormant state of micrometastases is involved in lack of nutrition and oxygen from vasculature in relation to angiogenic switch and/or the adaptive immune system [178–182]. Endothelial cells in the microenvironment can enhance dormant tumor cells via cell-to-cell interactions and induction of angiogenesis [180]. The ECM also plays an important role in activation of dormant cells. When tumor cells fail to adhere to the ECM, they may enter in dormancy. It has been postulated that micrometastases fail to properly connect to the ECM and survive in the dormant state because they are deprived of growth factors and angiogenic signaling. Adhesion to the ECM could induce tumor cells to switch a dormancy state to a proliferation state *via* integrin signaling [181]. On the other hand, both tumor cells and host stromal cells modulate the microenvironment such as ECM and vascular walls. Those mechanisms may regulate the maintenance in dormancy or the activation metastatic growth for a single tumor cell or micrometastases respectively (Figure 2) [17, 181].

*In vivo* molecular mechanisms of a variety of cancers including OS in dormant state have been assessed with genome transcriptional analysis [181]. This study suggests that antiangiogenic proteins such as angiomotin, which has been shown to suppress tumor growth and keep dormancy of tumor metastases [179], are upregulated during dormancy. Thus, the tumor proliferation and invasion are inhibited under preangiogenic state. Tumor cells in proliferation state also increased the key cancer pathways such as EGF receptor-1, IGF-IR, and PI3K. The mechanism of regulating tumor dormancy is unknown in OS. However, if it is possible to induce and/or keep in a dormant state or to induce cell death in residual dormant cells by targeting their survival and drug resistance mechanisms, the treatment for the patients with OS may be further improved.

## 10. Conclusion

OS associated with metastases still have poor clinical outcome, and conventional therapies are not fully effective. In addition, clinical output of novel available chemotherapeutic approaches is still unclear. Recent studies have disclosed new insights into the molecular mechanisms of metastasis as above mentioned. However, much more unknown questions remain; determinant factors of selective colonization in different organs, the mechanisms of tumor dormancy, and the mechanisms of metastasis suppressors, and so forth. Thus, future research critically needs to be directed towards identifying the molecular alterations in OS microenvironments.
